# Conveying facial expressions to blind and visually impaired persons through a wearable vibrotactile device

**DOI:** 10.1371/journal.pone.0194737

**Published:** 2018-03-27

**Authors:** Hendrik P. Buimer, Marian Bittner, Tjerk Kostelijk, Thea M. van der Geest, Abdellatif Nemri, Richard J. A. van Wezel, Yan Zhao

**Affiliations:** 1 Department of Biomedical Signals and Systems, MIRA Institute, University of Twente, Enschede, The Netherlands; 2 Department of Biophysics, Donders Institute, Radboud University, Nijmegen, The Netherlands; 3 VicarVision, Amsterdam, The Netherlands; 4 Department of Media, Communication, & Organization, University of Twente, Enschede, The Netherlands; 5 Department of Media and Design, HAN University of Applied Sciences, Arnhem, The Netherlands; University of Bath, UNITED KINGDOM

## Abstract

In face-to-face social interactions, blind and visually impaired persons (VIPs) lack access to nonverbal cues like facial expressions, body posture, and gestures, which may lead to impaired interpersonal communication. In this study, a wearable sensory substitution device (SSD) consisting of a head mounted camera and a haptic belt was evaluated to determine whether vibrotactile cues around the waist could be used to convey facial expressions to users and whether such a device is desired by VIPs for use in daily living situations. Ten VIPs (mean age: 38.8, SD: 14.4) and 10 sighted persons (SPs) (mean age: 44.5, SD: 19.6) participated in the study, in which validated sets of pictures, silent videos, and videos with audio of facial expressions were presented to the participant. A control measurement was first performed to determine how accurately participants could identify facial expressions while relying on their functional senses. After a short training, participants were asked to determine facial expressions while wearing the emotion feedback system. VIPs using the device showed significant improvements in their ability to determine which facial expressions were shown. A significant increase in accuracy of 44.4% was found across all types of stimuli when comparing the scores of the control (mean±SEM: 35.0±2.5%) and supported (mean±SEM: 79.4±2.1%) phases. The greatest improvements achieved with the support of the SSD were found for silent stimuli (68.3% for pictures and 50.8% for silent videos). SPs also showed consistent, though not statistically significant, improvements while supported. Overall, our study shows that vibrotactile cues are well suited to convey facial expressions to VIPs in real-time. Participants became skilled with the device after a short training session. Further testing and development of the SSD is required to improve its accuracy and aesthetics for potential daily use.

## Introduction

A wide range of daily life activities cause major problems for blind and visually impaired persons (VIPs), including wayfinding in unfamiliar surroundings, detecting objects and persons, and recognition of faces and facial expressions [1–4]. One such activity is face-to-face interaction: When they take place between two sighted people (SPs), much information is exchanged nonverbally via body posture, gestures, interpersonal proximity and facial expressions. For example, facial expressions are believed to be closely related to one’s emotions and provide information about the message one is trying to convey [5]. Because of their inability to fully access nonverbal information, VIPs who lost vision early in life can experience adverse effects on their social development, ultimately impacting their social inclusion as adults [3,6–8]. Despite demand from the VIP community [1,2], to our knowledge, there are only few assistive technologies available that attempt to support VIPs in accessing nonverbal communication in real time during social interactions.

In the absence of the ability to see, the human brain can learn to process information normally acquired through vision by using other senses, such as the auditory or haptic systems [9,10]. For VIPs, this means that information such as color, written information, nonverbal cues, or landmarks, can be obtained through auditory or haptic cues. The most well-known example is Braille, which is widely used amongst VIPs to interpret written information [9]. A study in the late 1960’s showed that it was possible to convey visual information to VIPs using a haptic display built into the back of a chair, a so-called sensory substitution device (SSD) [11]. This system, which translated visual information directly to haptic patterns, enabled VIPs (after extensive training) to pick up objects. More recently, several other SSDs were presented that use audio or tactile tongue displays to convey visual information through another sense [12–16]. However, in the case of conveying information during social interactions, the use of audio or a tactile tongue displays seem unsuited, for these concepts interfere with hearing and speech, which needs to be avoided during social interactions.

When it comes to conveying social information, it has been demonstrated that it is possible to convey spatial information (such as the location of and distance to other persons), walking directions, person identity, and social cues to VIPs through a vibrotactile belt, using variations in vibration location, frequency, and intensity [17–22]. Various studies have presented a tactile grid in the back of a chair to convey facial expressions (amongst others the Haptic Face Display (HFD) [8,23,24]). While the HFD conveyed information with a high level of detail (the device used 48 tactors to display 15 vibration patterns), it was not mobile, as it required users to sit in the chair for it to be effective [8,23]. Furthermore, a vibrotactile glove was developed to convey Ekman’s facial expressions of emotions plus neutral expressions through seven different vibrotactile patterns displayed on 14 tactors mounted on the back of the fingers [25]. In each of these studies, participants were quickly able to learn and interpret complex vibrotactile patterns conveyed. However, both studies focused on methods to convey information about facial expressions, but did not present a fully functional system that is capable of recognizing facial expressions and conveying these to its users in real time.

In this paper, a wearable SSD designed to support VIPs in determining the facial expressions of other persons is presented. The SSD classifies facial expressions into emotions, which are then conveyed using vibrotactile stimuli provided by a belt worn on the waist underneath clothing. Through user evaluations by VIPs and SPs, we sought to determine whether such a device could improve one’s ability to determine the facial expressions of others and whether such as device is desired for use by VIPs.

## Materials and method

### Participants

Medio 2016, VIPs who had participated in earlier studies, and lived at a reasonable distance from the University of Twente were approached to participate in the study. Ultimately, twenty participants were included in the study including 10 VIPs and 10 SPs (see Table 1 for an overview of the participant characteristics). To maximize the number of potential users, we choose to include a group of VIPs (age: 38.8, SD: 14.4, range = 18–58) with a wide range of visual impairments who reported difficulties recognizing facial expressions and consisted of both early and late blind persons. As a control, the SPs (age: 44.5, SD: 19.6, range = 20–68) were each gender and age matched to one of the VIPs, creating two groups with reasonably similar compositions. Exclusion criteria included other cognitive or sensory impairments besides visual loss. The study was approved by the ethical committee of the Faculty of Electrical Engineering, Mathematics and Computer Science of the University of Twente and conducted in accordance with the guidelines of the Declaration of Helsinki. Data acquired from the study were only used after obtaining oral informed consent from the participant. Participants were told they could quit participation at any moment, without having to provide a reason for doing so. There were no drop-outs after informed consent was obtained.

**Table 1:** Overview of the VIP participant characteristics.

Visually impaired group			
Gender	Age	Description of Vision Loss	Time of Occurrence
Male	27	Fully blind	Late blind
Female	18	Fully blind	Early blind
Male	58	Light perception	Congenitally blind
Female	23	Central vision loss: Stargardt Disease (macular degeneration)	Late blind
Female	44	Left eye: Light perception; Right eye: Tunnel vision	Late blind
Male	58	Blurred vision, Severe near-sightedness	Late blind
Female	50	Familial Exudative Vitreoretinopathy	Congenitally blind
Male	27	Peripheral tunnel vision	Early blind
Female	43	Left eye: Blurred vision; Right eye: Light perception, Glaucoma	Congenitally blind
Female	40	Light perception, Retinitis Pigmentosa	Early blind

### Apparatus

**Fig 1:**
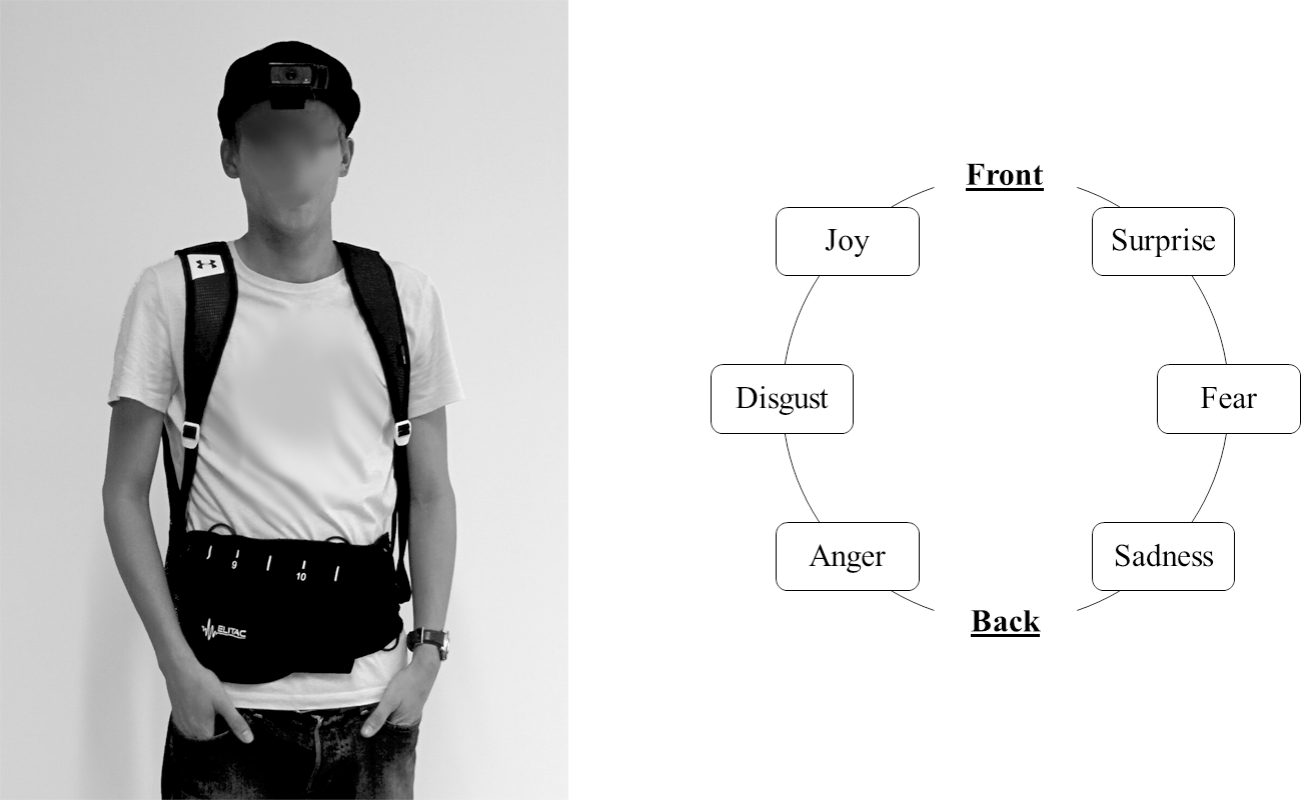
An overview of the sensory substitution system worn in the study. Left: Person wearing the device which consisted of a webcam mounted on a cap (1), a tablet in a mesh backpack (2), and an vibrotactile belt (3). Right: The six basic emotions and their placement on the vibrotactile belt worn around the waist of the user. More positive emotions were positioned in the front, whereas negative emotions were conveyed on the back.

The SSD used in the study is shown in Fig 1. The various components of the device were controlled and linked via custom software on a Microsoft Surface Pro 4 tablet (6th Gen 2.2-GHz Intel Core i7-6650U processor with Intel Iris graphics 540, Windows 10 operating system). Users wore a Logitech HD Pro Webcam C920 mounted on a baseball-cap to record images in the gaze direction. The detection of faces and facial expression recognition from this live video stream was achieved using FaceReader 6™ (Vicar Vision, Amsterdam, The Netherlands). This software uses a robust real-time face detection algorithm to detect a face from the video stream [26] and an artificial deep neural network that can classify facial expressions into one of six basic emotions (anger, disgust, joy, fear, surprise, sadness) as well as a neutral facial expression [5,27].

The detected facial expressions were conveyed to the user by a series of vibrating motors (tactors) which were connected to the tablet via a Bluetooth connection. These tactors (3V pancake direct current unbalanced motors with a maximum rotational speed of 150 cycles/s and maximum vibration strength of 158.3 ± 2.4 Hz), were attached to a fabric belt with Velcro worn around the waist (Science Suit, Elitac, Utrecht, The Netherlands). The waist was chosen as it is not often used for social interactions, unlike for example the hands. Furthermore, the waist provides sufficient space to place multiple tactors (sized 34 x 16 x 11mm) at the spatial distance required to ensure that people could easily distinguish vibrations from different tactors [28,29]. Vibrotactile signals on the torso can be distinguished with an acuity of 2 to 3 cm [30]; an even lower acuity can be achieved on the back near the spine, where it is likely that distances lower 1.3 cm are distinguishable [31]. The six tactors used in the current study were placed at least 4 cm apart, meaning the minimal distinguishable distances were amply complied with.

Each tactor was coupled to one of six basic emotions [5]. More positive emotions were positioned toward the front whereas negative emotions were positioned toward the back (see Fig 1 for tactor placement) in line with expressions of emotions (“butterflies in the stomach” or “stabbed in the back/talking behind one’s back” [32]). The one to one association of each tactor to an emotion was purposely chosen to make the task of learning and interpreting the vibrations very easy for VIPs. As the ultimate goal was for VIPs to use such a system in daily life situations during which they may face other sensory information and/or use other assistive devices that require their attention, the system was designed to avoid unnecessary sensory and cognitive overload in real-life situations.

Upon detection of a face, the user was alerted with two 150ms vibrations on all tactors with a 50ms break in between. After another 200ms, the tactor associated with the recognized facial expression vibrated so long as the expression held. This feedback was only provided if the facial expression detected deviated from the neutral expression. A long 300ms vibration on all tactors was used to indicate when the software no longer detected a face.

### Materials

Three types of materials were used as test stimuli to determine how accurately persons could identify facial expressions: pictures, silent videos, and videos with audio. The pictures and videos were derived from validated sets of pictures from the Warsaw Set of Emotional Facial Expression Pictures (WSEFEP) [33] and videos from the Amsterdam Dynamic Facial Expression Set (ADFES) [34], and included facial expressions of joy, surprise, fear, sadness, anger, and disgust [5]. Audio-visual stimuli were created by combining silent videos from the ADFES with (very obvious) annotated non-linguistic affect bursts (i.e. short bursts of sounds persons made while expressing an emotion) from other validated sets [35,36]. Although the set of Hawk and colleagues [35] did contain both audio and video files (not combined), video files from the ADFES were used due to their better image quality. Audio and video files were matched based on the annotated emotion and its intensity and combined using video editing software. All syncing was done manually to ensure the beginning of the facial expression and the affect burst matched.

To see how FaceReader performed when it was confronted with stimuli that were not directly loaded into the software, but that were subject to head movement and lighting conditions, only stimuli were used for which it was known that the software could detect the correct emotion. Therefore, prior to the experiments, all visual stimuli to be used in the experiment were loaded directly into FaceReader to verify that the software could determine the correct facial expressions in each of the stimuli under optimal conditions. Stimuli that were not correctly detected were excluded from the study. For comparison, earlier studies showed that the accuracy of SPs in determining facial expressions from these sets was 87% for the ADFES and 82% for the WSEFEP. Similarly, FaceReader achieved an accuracy of 88% and 89% for these sets, respectively [37].

### Experimental design

**Fig 2:**
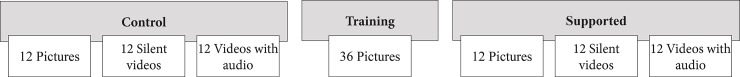
Study design. The experiment was divided into a control, training, and supported phase, each with 36 stimuli consisting of pictures, silent videos and videos with audio.

**Fig 3:**
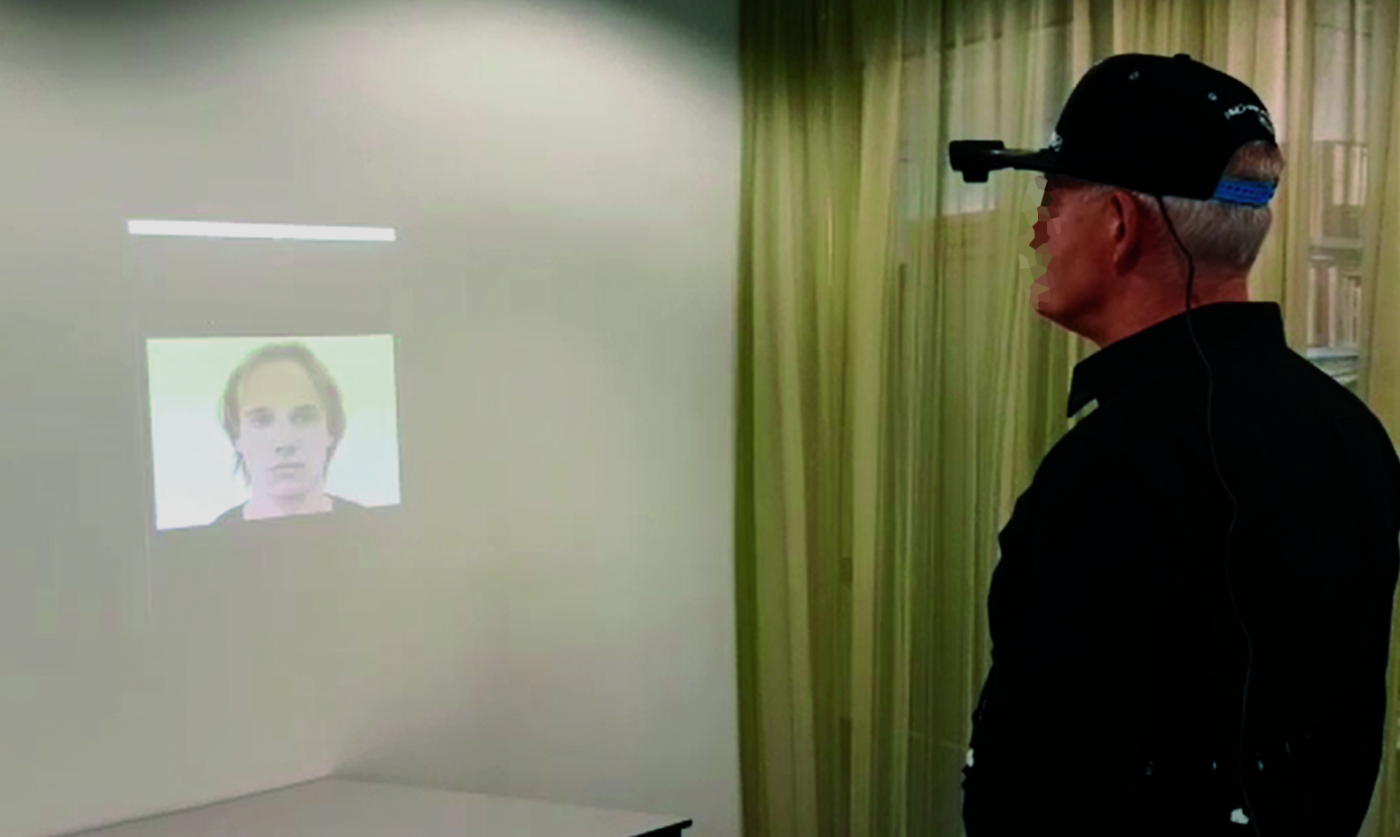
Experimental setup. The participant was positioned in front of a projector, which projected stimuli on a wall 2m away.

The experiment was divided into three phases (an unsupported control phase, a training phase, and a supported phase) (Fig 2). The visual stimuli were projected on a wall, two meters in front of the participant (Fig 3), while audio was played at a volume that all participants could clearly hear from the speakers of a laptop placed right behind the participant. The size of the projected face was slightly bigger than a normal face would be at a two-meter distance to create face sizes like those encountered in normal social interactions.

During the control and supported phases, 12 pictures, 12 silent videos, and 12 videos with audio were presented to the participant. The order in which stimuli were presented within each set of 12 was predefined, but randomized beforehand to ensure that participants could not guess which emotion was presented next. To avoid order bias, the stimuli sets were presented in reverse order for half of the participants (5 VIPs and 5 SPs). The training session included only pictures, the first 12 of which were presented in an order of emotion, whereas the remaining 24 were presented in a random order.

The control phase was used to ascertain how accurately subjects could identify the emotions displayed in the stimulus sets whilst relying only on their functional senses. A short beep was used to indicate when a stimulus was about to be presented, after which each stimulus was displayed for six seconds. Following each stimulus, the participants were instructed to indicate which emotion was expressed. The participants were made aware that no new stimulus would be displayed until they finished giving their answer. If a VIP was unable to detect the first three stimuli of a set, the session continued to the next set of stimuli.

During the training phase, which lasted for about 20 minutes to half-an-hour, participants were introduced to the SSD and received instructions on how to interpret the vibrotactile cues. After measuring waist circumference to ensure correct tactor spacing and placement, the minimum perceivable and maximum comfortable vibration strengths of each user were determined and the upper and lower boundaries of the tactor vibrations were programmed accordingly. Participants were then instructed which emotion was assigned to each tactor location. To familiarize participants with the device, three sets of 12 pictures were shown while they received the corresponding tactile cues on the belt. SPs were asked to close their eyes during training to ensure attention was directed to the vibrotactile cues. During the first 12 pictures, the participants were told which emotions were conveyed by the belt. For the second set of pictures, answers given by the participants were either confirmed or corrected by the examiner. For the final 12 pictures in the training set, participants practiced completely without receiving feedback.

In the last phase of the experiment, trained participants were supported by the device and asked to identify the emotions from a stimulus set consisting of 12 pictures, silent videos and videos with audio. In addition to the questions asked during the control measurements, participants were also asked to report the location of the vibrating tactor for each stimulus.

### Data analysis

Trials with correctly identified emotions were scored with a 1, whereas incorrect answers were scored with 0. For each measurement, mean performance scores were calculated. The performances of both the participant and FaceReader were rated in this way. For the user performance, it did not matter whether mistakes were made due to wrong interpretation of the vibrotactile cue by the respondent or to a misclassification by the FaceReader software causing the device to convey the wrong vibrotactile cue. The between-subject effects of group (SP or VIP), and the within-subject effects of phase (control—no SSD, supported—with SSD) and stimuli (pictures, silent videos, video with audio), were analysed using repeated measures ANOVA with an alpha of 0.05 in IBM SPSS Statistics 22. A similar analysis was also performed to analyse how participants performed when the FaceReader software misidentified the emotion shown. In this case, the between-subject effect of group (SP or VIP), and the within-subject effects of stimuli (pictures, silent videos, videos with audio) and FaceReader accuracy (wrong, correct) was analysed. Because data for some conditions were slightly skewed towards a performance 100%, the assumption of normality was violated, which should be considered when interpreting the results. Furthermore, Mauchly’s test was used to test the assumption of sphericity. If the assumption of sphericity was violated, the degrees of freedom were corrected using Greenhouse-Geisser or Huynh-Feldt corrections. *Post hoc* comparisons were performed using Bonferroni adjustments. Finally, the performance of the participants was compared to that of FaceReader to determine the extent the SSD contributed to the performance of the participants. Unless otherwise stated, descriptive statistics are represented by the mean±standard error of the mean.

## Results

**Fig 4:**
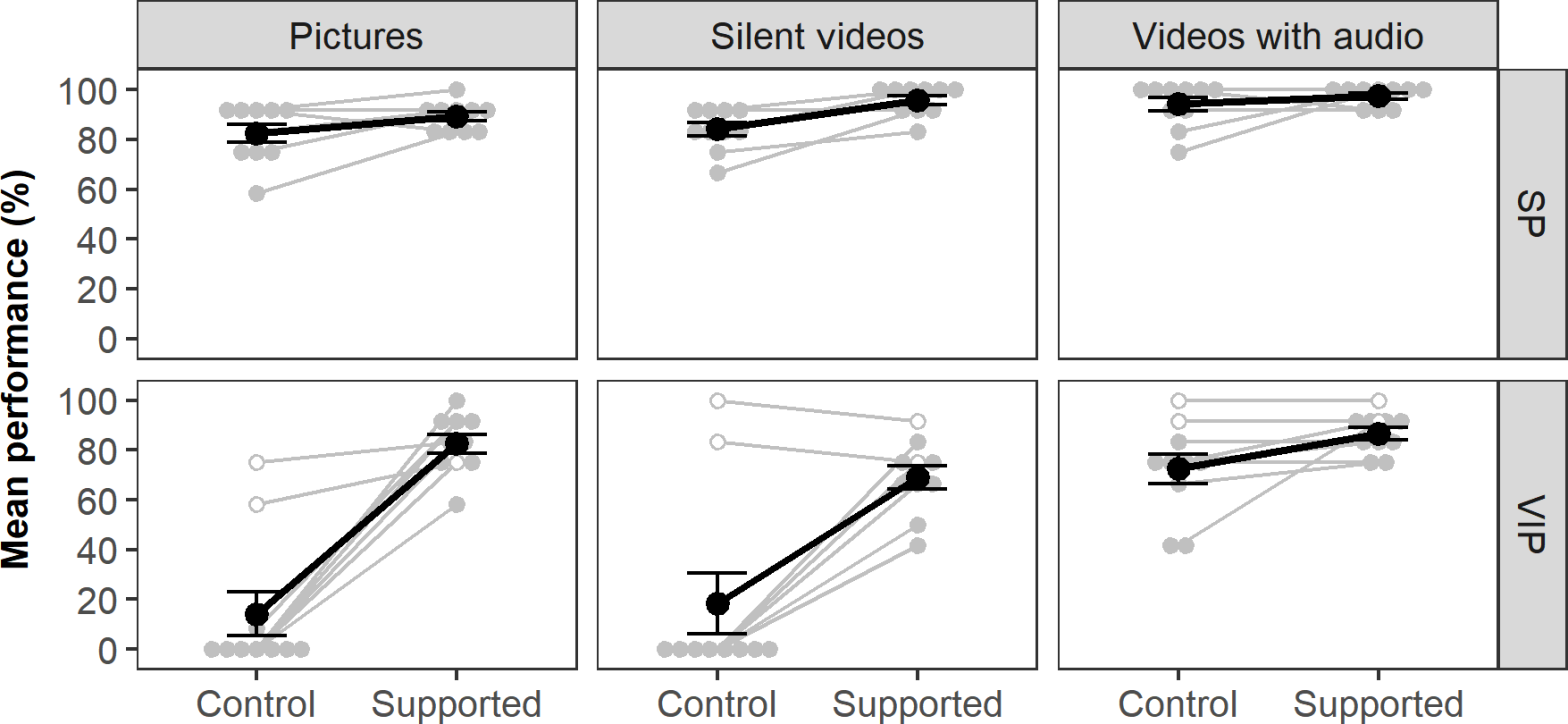
Scores for each phase, stimuli, and group. Black dots and lines are associated with the mean score of the subgroups whereas grey dots and lines are associated with individual participants. Grey dots with white filling were visually impaired participants who had sufficient remaining vision to detect stimuli unsupported. Error bars show standard error. SP: Sighted persons, VIP: visually impaired persons.

An overview of the performances for both participant groups under the different experimental phases can be found in Fig 4 and Table 2. Overall, higher performance levels were achieved with the support of the SSD compared to control for both SPs and VIPs across all types of stimuli. Statistical analysis showed significant main effects of phase, stimuli, and group on performance. Significant two-way interactions were found between phase and group, stimuli and group, and phase and stimuli. A significant three-way interaction also existed between phase, stimuli, and group.

**Table 2:** Overview of the mean performance across different phases, stimuli, and groups.

Group	Stimuli	Phase				
		Control		Supported		Difference
		Mean	SEM^a^	Mean	SEM^a^	
**VIP (N = 10)**	**All stimuli**	**35.0**	2.5	**79.4**	2.1	**44.4** *^b^
	Pictures	14.2	3.2	82.5	3.5	68.3 *** ^**b**^
	Silent videos	18.3	3.5	69.2	4.2	50.8 *** ^**b**^
	Videos audio	72.5	4.1	86.7	3.1	14.2 * ^**b**^
**SP (N = 10)**	**All stimuli**	**86.9**	1.8	**94.2**	1.2	**7.2**
	Pictures	82.5	3.5	89.2	2.8	6.7
	Silent videos	84.2	3.3	95.8	1.8	11.7
	Videos audio	94.2	2.1	97.5	1.4	3.3
**All participants (N = 20)**	**All stimuli**	**61.0**	1.8	**86.8**	1.3	**25.8** * ^**b**^
	Pictures	48.3	3.2	85.8	2.3	37.5 * ^**b**^
	Silent videos	51.3	3.2	82.5	2.5	31.3 * ^**b**^
	Videos audio	83.3	2.4	92.1	1.8	8.7 *** ^**b**^

The table presents the mean performance and standard error of the mean across different phases and stimuli for SPs and VIPs separately (top 2 sections) as displayed in Fig 4 and that for all participants combined (bottom section). In addition, the table also indicates the differences between the mean performances of the control and supported phases.

^a^ SEM = Standard error of the mean

^b^ *** p < .001, * p < .05.

### Effect of the SSD on performance

The SSD had a significant effect on performance (F(1,18) = 39.59, *p* < .001) for all participants combined: Average performance scores differed significantly (*p <* 0.001) between the supported phase (86.8±1.3%) and the control phase (61.0±1.8%). Note that Mauchly’s sphericity test was not applied for this within-subject factor, as there were only two levels (unsupported and supported)

In addition, there was an significant interaction effect for condition and group (F(1.18) = 20.55, *p* < .001): Whereas the mean score of VIPs increased significantly (*p* < 0.05) from 35.0±2.5% during control to 79.4±2.1% when supported; SPs also achieved an improvement (from 86.9±1.8% to 94.2±1.2%), but the difference was not statistically significant. These results suggested that participants were more capable of identifying facial emotions whilst supported by the SSD.

### Effect of sightedness on performance

The between-subject effect of group (SP or VIP) had a significant effect on performance (F (1,18) = 42.311, *p* < 0.001). The SPs were overall significantly better (*p* < 0.001) in detecting facial expressions than their VIP counterparts: the average performance across both phases was 90.6±1.1% for SPs and 57.2*±*1.8% for VIPs. Without the support of the SSD in the control phase, eight of the 10 VIPs could not identify emotions at all, while two VIPs were able to use their remaining vision to achieve performance scores above chance level. However, this sample size is too small and diverse (one person had tunnel vision in one eye and light perception in the other, whereas the other had only peripheral vision due to Stargardt disease) to conduct separate statistical analysis. The differences in accuracy between VIPs and SPs were much bigger in the control phase (35.0±2.5% for VIPs vs 86.9±1.8% for SPs) than in the supported phase (79.4±2.1% for VIPs vs 94.2±1.2% for SPs). In fact, the performance of VIPs when supported by the SSD reached a level that was not significantly different from those of SPs in the control phase (t(18) = 2.061, *p* = 0.054). Altogether these findings emphasize the beneficial effects of using the SSD for VIPs in recognizing emotions.

### Effect of stimulus type on performance

For the within-subject effect of stimulus, Mauchly’s test showed a violation of the assumption of sphericity (χ^2^(2) = 6.044, p < .05). Therefore, Greenhouse-Geisser corrections were applied (ε = .77). The type of stimulus presented to the participant (picture, silent videos, or videos with audio) had a significant effect on performance (F (1.54,27.71) = 50.259, *p* < 0.001). *Post hoc* tests showed that performance for videos with added audio (87.7±1.5% was significantly higher than for pictures (67.1±2.1%) and silent videos (66.9±2.2%). No significant performance difference was found between pictures and silent videos. Thus, participants found it easiest to identify emotions when additional auditory cues were provided.

For the two-way interaction between the type of stimuli and phase, Mauchly’s test showed that the assumption of sphericity was violated (χ^2^(2) = 7.817, p < .05) and Greenhouse-Geisser corrections were applied (ε = .731). A significant two-way interaction was found for phase and stimuli (F (1.46,26.30) = 23.05, *p* < 0.001). Furthermore, a significant three-way interaction effect between the phase, type of stimuli, and participant group was found (F (1.46, 26.30) = 16.35, *p* < 0.001). For SPs, there were no significant performance differences between control and supported phase for each type of stimuli. In contrast, VIPs showed significant performance improvements for pictures (68.3% increase, *p* < 0.001), silent videos (50.8% increase, *p* < 0.001), and videos with added audio (14.2% increase, *p* < 0.05) compared to control. In the control phase, VIPs were significantly better at determining the expressed emotions from videos with audio than those from other types of stimuli (*p* < 0.001). With the support of the SSD, the mean performance difference between pictures and videos with audio was no longer statistically significant. The difference between silent videos and videos with audio remained significant, possibly due to the inherent performance of FaceReader (see below). These results suggest that vibrotactile cues could enhance the recognition of facial expressions, especially in the absence of auditory cues.

### Interpretation of the vibrotactile signals

**Fig 5:**
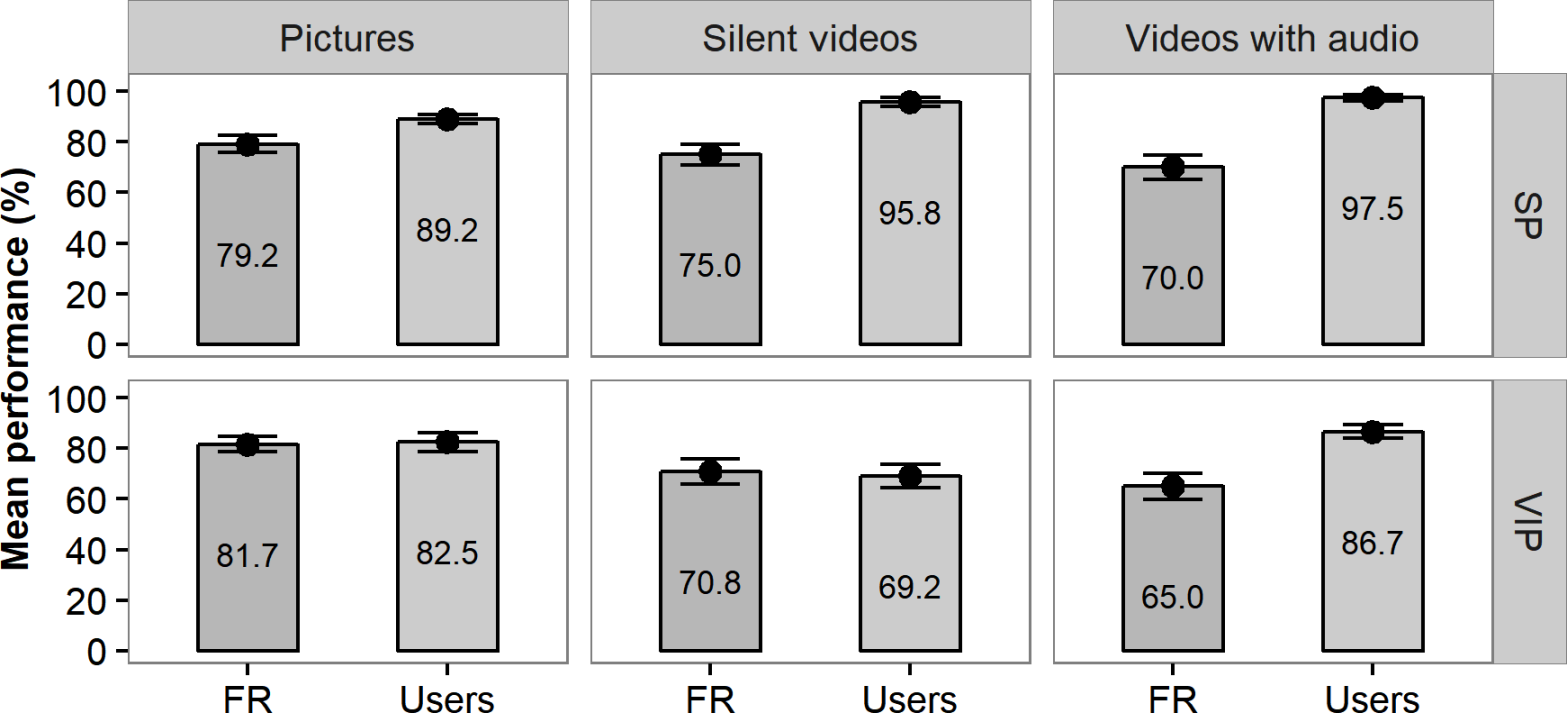
FaceReader accuracy versus user performance in the supported phase. This graph shows the mean performance in the supported phase of the participants (right, also shown in Fig 4 and Table 2) compared to FaceReader (left). Error bars represent standard error.

The accuracy of FaceReader was a limiting factor in the performance improvements of the VIPs. Overall, the software achieved an average accuracy of 73.6±1.6% in classifying the facial expressions from the experimental stimuli. SPs outperformed FaceReader overall, whereas VIPs performed only as well as FaceReader in absence of auditory cues whilst outperforming FaceReader (65% vs. 86.7%) when auditory cues were also provided (Fig 5).

**Fig 6:**
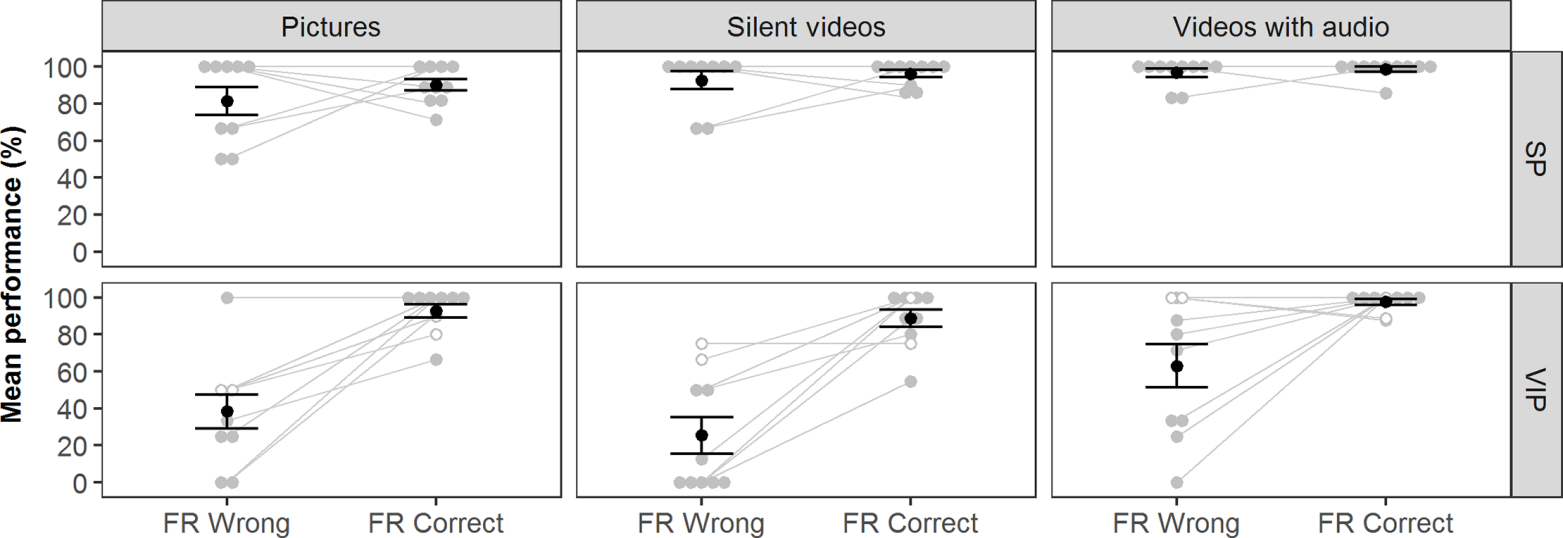
Performance of participants in recognizing facial expressions in relation to FaceReader accuracy. The average accuracy of SPs and VIPs when FaceReader (FR) correctly identified the facial expression compared to the performance if FaceReader misidentified the facial expression. Error bars represent the standard error. Grey dots with no filling correspond to VIPs who had sufficient remaining vision to detect stimuli unsupported.

To examine how participants dealt with inaccuracies in FaceReader, an additional analysis was conducted to compare how participants performed when FaceReader was correct with how they performed when FaceReader was incorrect (Fig 6 and Table 3). Mauchly’s test of sphericity showed that the assumption of sphericity is met for stimuli (χ^2^(2) = 1.275, p < .528) and the interaction between stimuli and FR (χ^2^(2) = 4.389, p < .111. Similar to before, the main effects of group (F (1,16) = 26.474, p < .001), stimuli (F (2,32) = 7.921, p < .01) and the two-way interaction between stimuli and group (F (2,32) = 3.752, p < .05 were significant. Notably, the main effect of FR (F (2,32) = 25.456, p < .001) and the interaction effect between FR and group (F (1,16) = 14.946, p < .002 were also significant. No significant interaction effects were found for the two-way interaction between stimuli and FR (F (2,32) = 3.194, ns) and the three-way interaction of stimuli, FR, and group (F (2,32) = 2.016, *ns*).

**Table 3:** Performance of participants in relation to FaceReader accuracy.

Group	Stimuli	FR	N	Performance Mean	SEM^a^	Mean difference
Sighted	Pictures	Wrong	25	84.00	7.48	14.2
		Correct	95	90.53	3.02	
	Silent videos	Wrong	30	93.33	4.63	3.6
		Correct	90	96.67	1.90	
	Videos with audio	Wrong	36	94.44	3.87	2.4
		Correct	84	98.81	1.19	
VIP	Pictures	Wrong	22	36.36	10.50	54.4 ***^b^
		Correct	98	92.86	2.61	
	Silent videos	Wrong	35	25.71	7.50	63.3 ***^b^
		Correct	85	87.06	3.66	
	Videos with audio	Wrong	42	66.67	7.36	34.6 *^b^
		Correct	78	97.44	1.80	

The table shows the mean and difference in mean participant performance, when FaceReader correctly and incorrectly detect the emotion shown. Furthermore, the table shows the number of times FaceReader was wrong/correct for each condition (N).

^a^ SEM = Standard error of the mean

^b^ *** p < .001, * p < .05.

The performance of SPs did not depend on the success of FR, which suggested that SPs were able to correct for the mistakes of FaceReader using visual and auditory cues. VIPs, however, performed significantly worse across all stimulus types when FaceReader conveyed the incorrect emotion. While unsurprising for stimuli lacking audio (pictures and silent videos), VIPs also performed significantly worse for videos with audio (66.7±7.4 vs 72.5±4.1). Although their performance for videos with audio exceeded that for pictures and silent videos, auditory cues were insufficient for VIPs to fully correct for FaceReader mistakes. VIPs seemed to be unable to correct for mistakes by the system using the auditory stimuli. Nevertheless, the performance of VIPs remained higher when the system was used in combination with auditory cues (86.7±3.1), because of the near perfect performance in cases where FR was correct (97.4±1.8).

## Discussion

The objective of the study was to determine the feasibility of using a wearable device to convey facial expressions of emotions through vibrotactile feedback. By combining various existing technologies, we developed a wearable SSD that conveys facial expressions to its users in real time through a vibrotactile belt. This study showed that participants could easily distinguish and interpret vibrotactile stimulation associated with the six basic emotions in real time. In fact, VIPs significantly improved their ability to determine facial expressions while wearing the SSD for all types of stimuli, reaching an overall accuracy of 79.4%. As participants were still able to use their senses of hearing and sight, if any, in determining the facial expressions, the SSD also did not interfere with other sensory modalities. Thus, our study confirms the conclusions of previous studies that haptic cues can be a beneficial tool for conveying visual information [8,18].

In line with earlier studies using the same annotated sets [33,34,37], SPs reached a performance mean of 86.9% without the help of the SSD. In the control phase, VIPs were able to determine the facial expression in 72.5% of the videos with audio, which is in line with the expected performance for the affects bursts [36]. Previous studies showed that the FaceReader software can recognize facial expressions from annotated sets of stimuli with an accuracy close to 90% [27,37]. In our study, the software reached lower accuracy averaging 73.6% (range: 65%-81.7%). This discrepancy may be explained by the fact that the stimuli presented in our study were not directly loaded into FaceReader, but rather fed from a live video stream, and therefore subject to head movements, changing focal length, and changes in lighting and luminance.

The results of the studies are consistent with the general principles of multisensory integration. According to the Bayesian view on multimodal cue integration, perception is probabilistic and in order to form a coherent percept of the world cues from different sensory modalities are combined in such a way as to favour the most reliable (or least uncertain) cues [38]. With limited sight, VIPs therefore generally rely on auditory and haptic cues (e.g. text-to-speech and braille). As shown in the study, VIPs achieved a high degree of accuracy in trials with auditory stimuli, even without additional haptic cues, since the auditory cues used in the study were very unambiguous and easy to interpret. The performance of VIPs to detect the correct emotion from auditory stimuli significantly improved as soon as (the even more unambiguous) haptic cues were added.

This improvement of performance was highly dependent on the accuracy of the software, as inaccuracies of the software were hardly corrected for by the VIP participants. Even when auditory stimuli were presented, mistakes by the software led to a significant decrease in performance, resulting in a performance that was lower than performance in auditory stimuli only. Overall however, the accuracy of the software was sufficient to improve the performance of VIPs for all types of stimuli, including auditory.

Furthermore, it is important to take into account that during social interactions in real-life conditions, emotions are often conveyed without auditory cues (e.g. smiling, frowning, etc.). In such cases, VIPs are forced to rely on the haptic cues conveyed by the vibrotactile device. It is promising that the performance of VIPs for videos with audio without haptic feedback was comparable to that for silent videos with the support of the vibrotactile belt, meaning that in the absence of auditory cues their ability to detect the correct emotion did not decrease. Moreover, the performance with both auditory and haptic feedback was higher than that with either sensory modality alone.

In contrast with previous SSD studies [8,11–16,21,22], the system presented here is fully wearable, does not interfere with other sensorimotor functions used in social interactions, namely touch of hand, speech, and hearing, and provides simple cues to represent a basic set of emotions to avoid cognitive overload in more realistic usage situations [39]. Furthermore, the full working prototype was tested in real time with VIPs, showing that the system could process live visual input and convey facial expressions of emotions in an easily interpretable fashion.

The responses to the device were generally positive amongst the VIPs. VIPs described various scenarios in which the device could be beneficial including face-to-face and group meetings (in line with [21]) and were willing to try the device in such settings. Nevertheless, the participants stated that the device required some alterations before they would use it over an extended period. Participants namely had reservations about the weight and fit of the cap-mounted camera and were concerned that the SSD would bring unwanted attention to their impairment. Finally, to make it more worthwhile for VIPs to wear the device for the entire day, the participants desired additional features such as those within the domain of social interactions or beyond, such as outdoor navigation or the access to public transport information [1,2,4,40].

### Limitations of the study

One could argue that it is perhaps unsurprising that VIPs were able to learn how to use the SSD within a short training period and achieve significant performance improvements, considering results from earlier studies [30,31]. Indeed, only six tactors placed at least 4 cm apart were used on the waist, while the spatial acuity of the torso is between 2 to 3 cm [30]. Moreover, participants were only required to learn one to one associations between six tactors and emotions, while earlier research has shown that persons are able to learn far more complex (vibrotactile) cues [8,9,11,12,15]. Nevertheless, the ultimate goal was to create a system that VIPs could easily use in real-life situations. According to [41,42], the ability to process tactile information is likely to be significantly worse in real-world conditions where other sensory inputs are competing for attention. The simplicity of the system and the sensory mapping was therefore intentional lest the vibrotactile cueing becomes unnecessarily difficult in daily life.

Another drawback of the study is its potential lack of generalizability to real-life situations, a concern that was also raised in [41,42]. In the experimental setup, the stimuli were presented on a fixed position and participants were instructed as to where the stimuli were presented. In real-life, users would have to localize and aim the camera towards the targeted person on their own. The lighting and gaze direction of the conversation partner were also stable in the experimental setup but would change continuously in the real world, thus impacting the quality of the analysis of facial expressions. Furthermore, it is important to thoroughly determine how well device users can interpret the vibrotactile cues in real-life situations, where other sensory stimuli might compete for attention and cognitive overload might become an issue. Second, there was a purposeful 550ms delay between the displayed stimulus and the vibrotactile cues associated to the displayed facial expression to warn users that a face was recognized. This caused for the fact that audio and visual cues were often interpreted before vibrotactile information was conveyed. In such cases the vibrotactile cues were merely used to confirm or adjust already made decisions and caused more confusion than clarity.

### Conclusions

This study showed that a SSD like the one presented, using vibrotactile cues at the waist, was a feasible method to convey information about facial expressions to VIPs, which may lead to improvements in their social interactions [2,18]. Participants were quickly able to learn how to interpret the cues conveyed by the device and combined this with information acquired from other functional senses. Furthermore, VIPs saw potential use of the device in real situations. Nevertheless, for the device to be readily adopted and accepted by VIPs as a daily life assistive technology, a more aesthetically pleasing design is required (e.g. smaller camera, less weight, unobtrusiveness) and more usage goals should be addressed (e.g. navigation). Finally, studies that are more closely resembling realism are needed to determine the accuracy of the device in real-life situations and user acceptance of the technology over time.

## Supporting information

S1 Dataset Performance data. The data that was used for analysis.
(XLSX)

S1 Related work Enhancing emotion recognition in VIPs with haptic feedback. Extended abstract presented as a poster at the 18th International Conference on Human-Computer Interaction, 17–22 July 2016, Toronto, CA.
(PDF)
