# Supplementary material for: Conveying facial expressions to blind and visually impaired persons through a wearable vibrotactile device
Source: PLoS One. 2018 Mar 27;13(3):e0194737. doi: 10.1371/journal.pone.0194737 (PMC5870993; doi:10.1371/journal.pone.0194737)
Supplement: S1 Related work — Extended abstract presented as a poster at the 18th International Conference on Human-Computer Interaction, 17–22 July 2016, Toronto, CA. (PDF) [file pone.0194737.s002.pdf]

Enhancing emotion recognition in VIPs with haptic feedback

Hendrik Buimer [1], Marian Bittner [1], Tjerk Kostelijk [2], Thea van der Geest [3], Richard van Wezel [1,4], Yan Zhao [1]

1 Department of Biomedical Signals & Systems, MIRA Institute, University of Twente, 2 VicarVision, 3 Department of Media, Communication, & Organization, University of Twente, 4 Biophysics, Donders Institute, Radboud University Nijmegen

Abstract. The rise of smart technologies has created new opportunities to support blind and visually impaired persons (VIPs). One of the biggest problems we identified in our previous research on problems VIPs face during activities of daily life concerned the recognition of persons and their facial expressions. In this study we developed a system to detect faces, recognize their emotions, and provide vibrotactile feedback about the emotions expressed. The prototype system was tested to determine whether vibrotactile feedback through a haptic belt is capable of enhancing social interactions for VIPs.

The system consisted of commercially available technologies. A Logitech C920 webcam mounted on a cap, a Microsoft Surface Pro 4 carried in a mesh backpack, an Elitac tactile belt worn around the waist, and the VicarVision FaceReader software application, which recognizes facial expressions.

In preliminary tests with the systems both visually impaired and sighted persons were presented with sets of stimuli consisting of actors displaying six emotions (e.g. joy, surprise, anger, sadness, fear, and disgust) derived from the validated Amsterdam Dynamic Facial Expression Set and Warsaw Set of Emotional Facial Expression Pictures with matching audio by using nonlinguistic affect bursts. Subjects had to determine the emotions expressed in the videos without and, after a training period, with haptic feedback.

An exit survey was conducted aimed to gain insights into the opinion of the users, on the perceived usefulness and benefits of the emotional feedback, and their willingness of using the prototype as assistive technology in daily life.

Haptic feedback about facial expressions may improve the ability of VIPs to determine emotions expressed by others and, as a result, increase the confidence of VIPs during social interactions. More studies are needed to determine whether this is a viable method to convey information and enhance social interactions in the daily life of VIPs.

Keywords: Sensory substitution, wearables, user-centered design

adfa, p. 1, 2011.
© Springer-Verlag Berlin Heidelberg 2011

1 Introduction

The rise of smart technologies, such as smartphones, smartwatches, and other wearables has led to new ways of conveying information to users. In the current paper, we present a wearable system to support visually impaired persons (VIPs). Previously, interviews were conducted amongst VIPs in The Netherlands to determine the biggest problems and challenges they face in daily life, after which a survey was held to rank activities of daily life on both perceived difficulty and perceived importance. One of the issues addressed, and ranked highest on difficulty in the survey, was the recognition of persons [1]. An example of such problems is the inability to determine facial expressions which others are displaying during social interactions [2]. Therefore, we developed an assistive aid, based on the notion of sensory substitution, to support VIPs with the recognition of facial expressions of others.

Sensory substitution, in which real world information is transferred to the human sensory interface by using artificial sensors, is not a new development, with Braille (developed in the 19th century) being one of the most famous examples to date [3]. In the late 60's Bach-y-Rita proposed that sensory substitution is possible, and dependent on available artificial receptors [4]. Later studies indeed demonstrated that the human brain has the ability to adapt to artificial receptors through a tactile machine interface [3]. In other words, with the right artificial receptors, it should be possible to give VIPs visual information by conveying it through another sense.

Several studies have proven that haptic feedback around the waist can be used to convey specific information, such as interpersonal distance or locations of persons, to visually impaired users [2, 5, 6]. However, to our knowledge, no one has tried to convey facial expressions and associated emotions through such a system.

To create new assistive technology for the blind and visually impaired we linked existing technologies. A Logitech C920 webcam, mounted on a cap, was used to capture images of the conversation partner of the user. FaceReader software (VicarVision, Amsterdam), which ran on a Microsoft Surface Pro 4, then analyzed the images in real time and categorized the facial expressions displayed as one of six basic emotions (e.g. anger, disgust, fear, joy, sadness, surprise) [7]. The emotion recognized by the software was conveyed using a haptic belt with six tactors, worn around the waist (Elitac, Utrecht). Each tactor was assigned to one emotion, whereas vibration intensity represented the intensity with which an emotion was displayed according to the software (see Figure 1). A more detailed description can be found in the method section.

In the current study we want to determine to what extent tactile-vision sensory substitution by means of vibrotactile feedback through a belt around the waist can support VIPs' ability to determine facial expressions and related emotions of conversation partners.

Fig. 1. Location of the tactors and the emotion assigned to it across the waist.

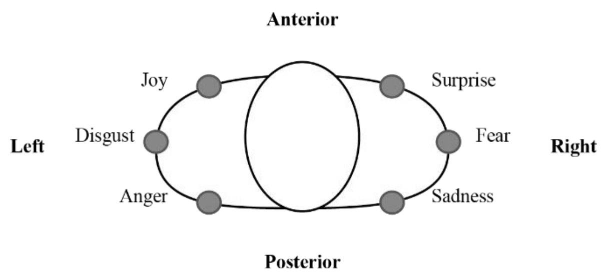

2 Method

2.1 Apparatus

The system consists of a webcam, a tablet running FaceReader software, and a haptic belt. The FaceReader software, running on the tablet, analyzes webcam content approximately at a rate of 5-6 frames per second. The software finds a face, models it, and then classifies the facial expressions of the face into one of seven emotions, which are: neutral, anger (*angry*), disgust (*disgusted*), fear (*scared*), joy (*happy*), sadness (*sad*), and surprise (*surprised*) [8-13].

This information is then conveyed through an Elitac haptic belt that was connected to the tablet by means of a Bluetooth connection. In the current setup six tactors are used to convey emotion information. On recognition of a face, all tactors on the belt activate at the same time with two bursts of 150ms and a break of 50ms in between. 200ms after the two bursts, the tactor associated to the displayed facial expression, as recognized by FaceReader, will vibrate as long as a face is detected. The intensity of the displayed emotion determines the intensity of the vibration, which has 15 levels of intensity. In case of a neutral facial expression, no tactors are activated. Once a face is no longer detected, the tactors will vibrate for 300ms.

2.2 Subjects

Preliminary tests were performed with one blind and three fully sighted subjects. Besides visual impairments, subjects included in the study had no other sensory or cognitive impairments. All subjects were in their 20s and were university students.

2.3 Materials

Each subject was confronted with 108 stimuli (60 pictures, 24 videos without audio, and 24 videos with audio), equally representing six basic emotions [7]. Validated sets of actors displaying emotions were used: 48 videos from Amsterdam Dynamic Facial Expression Set [14]; 60 pictures from the Warsaw Set of Emotional Facial Expression Pictures [15]. Due to the fact that both the pictures and the videos were not accompanied

with audio, audio was added to half of the videos, using two validated sets of nonlinguistic affect bursts [16, 17]. The affect bursts and videos were matched based on the emotions expressed and the intensity of the emotion, creating a stimulus that was as natural as possible. Before the experiment, all the stimuli were analyzed with the FaceReader software; only stimuli for which FaceReader was able to determine the emotion were included during testing.

2.4 Psychophysical testing

Testing was divided into three phases (baseline, training, and experiment) and lasted about one and an half hour with short breaks in between. During the baseline phase 36 different stimuli, consisting of 12 pictures, 12 videos without audio, and 12 videos with audio were shown to the subject without additional feedback (Figure 2). Each basic emotion was represented 6 times per phase. Before each stimulus the subjects heard a beep to indicate a new facial expression will be shown for about six seconds. After the end of each stimulus the subject was asked to indicate whether the displayed emotion was positive of negative, which emotion was displayed, the intensity of the emotion, and on which sensory input their conclusion was based. The stimuli lacking audio were also briefly shown to the visual impaired in order to acquaint them with the setup of the study.

The training phase was used to allow the subjects to familiarize themselves with the vibrotactile system. First, the desirable minimum and maximum vibration intensity, and the just notable difference between vibrations were determined. After calibrating the belt, the sighted subjects were blindfolded. The examiner gave a brief explanation on which tactors were assigned to each emotion. Next, the subjects were shown 12 pictures sorted by emotion and were told what emotion was displayed. Afterwards, 12 pictures were shown in a random order of emotions after which the subject reported which emotion they thought were displayed in the stimulus. The examiner either corrected or confirmed the answer the subject gave. Finally, the subject was shown 12 more pictures in a random order to train without confirmation from the examiner.

During the experiment phase, subjects were asked to determine the emotions in the stimuli using the system relying on all their available senses, as well as the vibrotactile belt. The procedure of the experiment phase is the same as the baseline phase. However, different stimuli were used and the subject was additionally asked to indicate after each stimulus the location where the vibration was felt.

After the three phases the subject completed a short questionnaire on how information was conveyed (example statement: “I could easily determine which tactor was vibrating”), on a five-point Likert scale (1 totally disagree, 2 disagree, 3 neutral, 4 agree, and 5 totally agree). In addition, open questions were asked about the ease of use of the system and whether the subject saw potential in the system for future usage.

Fig. 2. Study design

Baseline phase	Training phase	Experiment phase
12 Pictures	12 Pictures	12 Pictures
12 Videos without audio	12 Pictures	12 Videos without audio
12 Videos with audio	12 Pictures	12 Videos with audio

3 Preliminary results

The preliminary tests with three sighted subjects and one visually impaired user showed that subjects were fairly quick in learning to interpret the information conveyed by the vibrotactile belt. In the pre-test a 21 year old sighted respondent stated "it was difficult to understand in the beginning, but once you know where everything is located you can quickly learn to interpret the system." The system even assisted a sighted subject when the displayed emotion was ambiguous: "I noticed I was looking for confirmation by the system […], in some cases I found it difficult to decide, and then the system recognized anger, so I agree with the system (24 year old, sighted female, pre-test)."

The first visually impaired subject was a 27 year old fully blind male university student. In the experiment phase he correctly interpreted 66.7% of the emotions displayed. He was able to successfully determine 83.3% of the conveyed emotions by the vibrotactile belt, as detected by FaceReader (correctly interpreted emotions displayed in the validated set in 75% of the stimuli). Considering the fact that it was physically impossible for him to detect any emotion using the non-audio stimuli without the system, this is a major improvement. For the audio stimuli, the subject slightly improved, agreeing on nine out of twelve times without the system and eleven out of twelve times while wearing the system. A schematic overview of the scores by the visually impaired user can be found in table 1.

Table 1. Agreement between validated sets and the visually impaired user

Baseline phase			Training phase	Experiment phase		
**P**	**VWOA**	**VWA**		**P**	**VWOA**	**VWA**
0%	0%	75%		58.3%	50%	91.7%

Note. P = pictures, VWOA = videos without audio; VWA = videos with audio

Whereas the sighted subjects saw potential for VIPs rather than for themselves, the visually impaired subject stated that he does not expect to use a specific system for emotion recognition –as it is now- on a daily basis: "I think that if I could use it only for this purpose [emotion detection], I would not use it that quickly." However, he stated that he saw future potential for the system as a whole: "A belt like this could be worn for multiple reasons, such as a navigation aid. And when you are wearing the system already, how difficult could it be to turn on an app to recognize emotions once you are in a meeting? I think I would use it then, and I am sure I will try it out, just of curiosity."

In general, the subjects were positive about the system. The tactors were well placed and the subjects were able to distinguish vibrations originating from different units. The vibration intensity and associated sounds were acceptable in the current situation. However, the visually impaired subject stated that the sounds might be annoying in situations where everyone is rather quiet, such as in work related meetings.

4 Discussion

In the current study, we investigated whether tactile-vision sensory substitution by means of vibrotactile feedback through a belt around the waist can help people to determine the facial expressions and recognize emotions of their conversation partners. The preliminary results would seem to suggest that this is indeed possible with the system developed.

Although the system can detect faces and classify emotions reasonably well, its performance is impacted by lighting conditions and contrast of the test environment. FaceReader was capable of determining the correct facial expressions when videos were directly loaded into the software. However, during the actual experiment, where faces presented in a real time video feed were analyzed, FaceReader was less successful.

Thus, a possible limitation of the study is a lack of generalizability to real life situations. The study contents and procedure were setup to investigate whether both visually impaired and sighted persons were able to use the system and interpret the information conveyed through vibrotactile feedback in a controlled setting, using validated facial expressions stimuli, artificial lighting conditions, and a limited set of facial expressions consisting of six basic emotions. Therefore, it remains unknown how well the system will perform in real life, with ever changing lighting conditions and a wider range of facial expressions than the six basic emotions used in this study.

We plan to expand the current study with more subjects, after which we will investigate how the system and its users behave in real life situations. The current system is not always accurate in detecting the right emotion in suboptimal lighting conditions and lacks consistency. While sighted people can correct for these errors by using their sight, it is interesting to study how VIPs cope with such flaws in the system.

While the information conveyed by the system was limited to six basic emotions, the software used is capable of providing much more detailed information, based on a circumplex model that combines valence and arousal. Therefore, it should be further investigated how the amount of information conveyed though the haptic belt can be increased in order to cover a greater range of emotions.

Finally, it is interesting to investigate whether sensory substitution occurs over time and if users of the system unconsciously interpret the information conveyed by the system, thus enabling VIPs to actually feel others' emotions.

In conclusion, there are indications that users are able to quickly learn and interpret the information conveyed through the haptic belt. The system presented in this paper seems to confirm Bach-y-Rita and Kercel's predictions considering the possibility to substitute functions of the human sensory system with artificial receptors as long as those are up for the task [3]. We believe, the system presented can support VIPs with

the recognition of facial expressions and associated emotions, while showing great potential for expansion to other functionalities, e.g. detecting gestures and gaze direction, socially accepted behavior, and navigation tasks.

5 References

1. Van der Geest, T.M. and H.P. Buimer. *User-centered priority setting for accessible devices and applications.* in *Mensch und Computer 2015*. 2015. Stuttgart: De Gruyter Oldenbourg.
2. Krishna, S., et al., *A systematic requirements analysis and development of an assisitive device to enhance the social interaction of people who are blind of visually impaired*, in *Computer Vision Applications for the Visually Impaired*. 2008: Marseille, France.
3. Bach-y-Rita, P. and S.W. Kercel, *Sensory substitution and the human-machine interface.* TRENDS in Cognitive Sciences, 2003. **7**(12): p. 541-546.
4. Bach-y-Rita, P., *Sensory Plasticity: Applications to a Vision Substitution System.* Acta Neurologica Scandinavica, 1967. **43**: p. 417-426.
5. McDaniel, T., et al., *Using a haptic belt to convey non-verbal communication cues during social interactions to individuals who are blind*, in *HAVE 2008*. 2008: Ottawa.
6. McDaniel, T., et al., *Heartbeats: A methodology to convey interpersonal distance through touch*, in *CHI*. 2010: Atlanta, Georgia, USA.
7. Ekman, P., *An argument for basic emotios.* Cognition and Emotion, 1992. **6**(3/4): p. 169-200.
8. Bishop, C.M., *Neural Networks for Pattern Recognition*. 1995, Oxford: Clarendon Press.
9. Cootes, T. and C. Taylor, *Statistical models of appearance for computer vision.* 2000.
10. Viola, P. and M. Jones, *Robust Real-time Face Detection.* International Journal of Computer Vision, 2004. **57**(2): p. 137-154.
11. Van Kuilenburg, H., M. Wiering, and M.J. Den Uyl. *A Model Based Method for Automatic Facial Expression Recognition*. in *16th European Conference on Machine Learning*. 2005. Porto, Portugal: Springer-Verlag, GmbH.
12. Den Uyl, M.J. and H. Van Kuilenburg. *The FaceReader: Online Facial Expression Recognition*. in *Measuring Behaviour*. 2005. Wageningen, The Netherlands.
13. Van Kuilenburg, H., et al. *Advances in face and gesture analysis*. in *Measuring Behavior*. 2008. Maastricht, The Netherlands.
14. Van der Schalk, J., et al., *Moving faces, looking places: Validation of the Amsterdam Dynamic Facial Expression Set (ADFES).* Emotion, 2011. **11**(4): p. 907-920.
15. Olszanowski, M., et al., *Warsaw set of emotional facial expression pictures: a validation study of facial display photographs.* Frontiers in psychology, 2015. **5**: p. 1516.

16. Hawk, S.T., et al., *"Worth a Thousand Words": Absolute and Relative Decoding of Nonlinguistic Affect Vocalizations.* Emotion, 2009. **9**(3): p. 293-305.
17. Lima, C.F., S.L. Castro, and S.K. Scott, *When voices get emotional: A corpus of nonverbal vocalizations for research on emotion processing.* Behavioural Research Methods, 2013. **45**: p. 1234-1245.
